# Pericytes and the Control of Blood Flow in Brain and Heart

**DOI:** 10.1146/annurev-physiol-031522-034807

**Published:** 2023-02-10

**Authors:** Thomas A. Longden, Guiling Zhao, Ashwini Hariharan, W. Jonathan Lederer

**Affiliations:** 1 Department of Physiology, University of Maryland School of Medicine, Baltimore, Maryland, USA; 2 Laboratory of Neurovascular Interactions, Center for Biomedical Engineering and Technology, University of Maryland School of Medicine, Baltimore, Maryland, USA; 3 Laboratory of Molecular Cardiology, Center for Biomedical Engineering and Technology, University of Maryland School of Medicine, Baltimore, Maryland, USA

**Keywords:** pericytes, heart, brain, metabolism, neurovascular coupling, electro-metabolic signaling, ion channels, calcium, potassium

## Abstract

Pericytes, attached to the surface of capillaries, play an important role in regulating local blood flow. Using optogenetic tools and genetically encoded reporters in conjunction with confocal and multiphoton imaging techniques, the 3D structure, anatomical organization, and physiology of pericytes have recently been the subject of detailed examination. This work has revealed novel functions of pericytes and morphological features such as tunneling nanotubes in brain and tunneling microtubes in heart. Here, we discuss the state of our current understanding of the roles of pericytes in blood flow control in brain and heart, where functions may differ due to the distinct spatiotemporal metabolic requirements of these tissues. We also outline the novel concept of electro-metabolic signaling, a universal mechanistic framework that links tissue metabolic state with blood flow regulation by pericytes and vascular smooth muscle cells, with capillary K_ATP_ and Kir2.1 channels as primary sensors. Finally, we present major unresolved questions and outline how they can be addressed.

## INTRODUCTION

**Figure 1:**
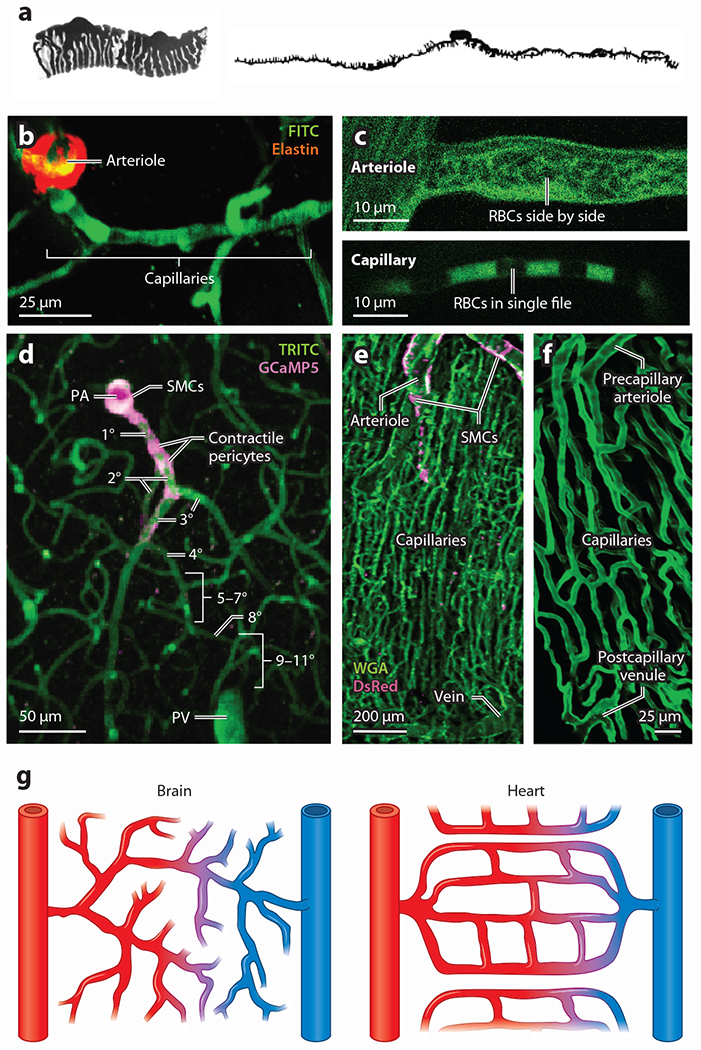
Microcirculation in heart and brain. (*a*) Original illustrations of pericytes by Zimmermann depicting two pericytes of a precapillary arteriole in cat kidney (*left*) and a thin-strand pericyte observed on a human capillary (*right*). Panel adapted with permission from Reference 3; copyright 2022 Springer Nature. (*b*) An arteriole-capillary transition in brain in vivo showing elastin staining (*red*) of the penetrating arteriole, which abruptly ends at the point of transition into the capillaries. (*c*, *top*) In arterioles, RBCs (dark objects silhouetted against green, fluorescent plasma) tumble past one another side by side due to the wider diameter of the vessel. (*c*, *bottom*) In capillaries, RBCs squeeze to enter to narrow confines of the vessel and pass in single file. (*d*) A PA in a mouse brain giving way to highly branching capillaries. In brain, the capillaries (*green*) can be numbered by branch order, with the assigned number increasing by one with each successive branch point. Pink fluorescence reflects the presence of the Ca^2+^ indicator GCaMP5, which is expressed under the control of the *Acta2* promoter. This highlights both SMCs of the PA and the contractile pericytes of the initial branches of the capillary bed. (*e*) Confocal image showing the high density of capillaries in heart. DsRed, under the NG2 promoter (*pink*), is highly expressed in arteriole smooth muscle cells but not in veins, which makes the feeding arteriole and vein readily distinguishable. (*f*) Zoomed-in image showing the capillary hierarchy in heart bookended by a precapillary arteriole and postcapillary venule. In heart, the precapillary arteriole and capillaries are readily distinguished by their different sizes and different orientations with myocytes. Note the high degree of looping anastomoses compared to brain. (*g*) Simplified illustrations highlighting the major differences between heart and brain circulatory organization. In brain (*left*), the capillary branching pattern appears to be somewhat random, branches give way to capillaries that head into the parenchyma in all directions, and anastomoses are much less frequent in this context. In heart (*right*), the capillaries form regular anastomoses, giving rise to loops that pass through and around adjacent cardiomyocytes. Abbreviations: FITC, fluorescein isothiocyanate; NG2, neuronal-glial antigen 2; PA, penetrating arteriole; PV, penetrating venule; RBC, red blood cell; SMC, smooth muscle cell; TRITC, tetramethylrhodamine isothiocyanate; WGA, wheat germ agglutinin.

Pericytes were first observed and documented by Eberth (1) and Rouget (2) approximately 150 years ago, and the term pericyte (surrounding cell, from the Greek prefix *peri*- denoting something that is around or nearby) was coined by Zimmermann a century ago (3). Even without modern imaging methods, the illustrations in these early works were able to capture the beauty and diversity of pericyte morphology with stunning detail and accuracy (Figure 1a). This diversity has both intrigued and confounded physiologists attempting to understand pericyte function since their discovery and continues to be a source of considerable controversy. However, advances in genetic engineering, imaging, and molecular profiling are now enabling studies that probe the functions of the cells that compose the pericyte continuum with unprecedented depth and precision, and the recent exponential increase in interest in pericyte biology promises to rapidly advance our future understanding.

The provocative positioning of pericytes on the outer wall of capillaries—their processes and cell bodies making intimate contact with adjacent capillary endothelial cells (cECs)—prompted early speculation that they play roles in controlling blood flow (4). In the intervening years, mounting evidence has confirmed this, although we still lack a detailed mechanistic understanding of these processes. Studying the functions of pericytes in organ systems with distinct metabolic requirements and different blood flow profiles may not only underscore commonalities of pericyte structure and function but also provide opportunities to uncover specializations that have evolved to equip these cells with the ability to regulate blood flow according to specific local energy demands. Accordingly, here we focus our attention on the emerging roles of pericytes in blood flow control in both heart and brain in health and disease.

As the primary pump driving blood around the body, the heart must work consistently throughout an animal’s lifetime to ensure uninterrupted delivery of oxygen (O_2_) and nutrients. Cardiac cells beat continuously, with their pace—and therefore energy requirements—modulated by external factors such as sympathetic:parasympathetic balance, hormone levels, and overwhelmingly the degree of whole-animal activity (5–8). Intrinsic local control of cardiac blood flow operates under the control of an important electro-metabolic signaling (EMS) mechanism, first identified in our recent work and in which pericytes play a vital role that is in the process of being defined (9, 10). The brain, in contrast to heart, exhibits variations in activity and energy demand across broader spatiotemporal scales, dictated by the computational demands that are placed upon neuronal networks spanning local and interregional territories at any given time. Blood flow in this context is continually adjusted to meet these highly fluctuating energy requirements through the process of neurovascular coupling (11, 12), where the cells of the vasculature modulate blood flow via the engagement of local signaling cascades in response to increased neural activity. A primary form of local blood flow control in brain is exerted by parenchymal potassium concentration ([K^+^]), which fluctuates according to neuronal activity (13). K^+^ controls the activity of the strong inward rectifier K^+^ channel, Kir2.1, which plays an important functional role in cECs (14, 15). In both heart (10) and brain (14, 15), this feature of cECs lays the foundation for rapid electrical signaling throughout the vasculature to regulate blood flow. A critically important emerging feature of both heart and brain pericyte physiology is that these cells can communicate electrically through gap junctions with underlying cECs to modulate this ongoing electrical activity (9, 10, 16–19).

Recent reviews (20, 21) have focused broadly and extensively on the multitude of molecular mechanisms through which pericytes may control blood flow. Here, our focus is on what can be learned from pericytes operating in two different organ systems with high energy requirements, and in particular, their potential roles in energy sensing and electrical signaling to control blood flow. We begin by reviewing the challenges associated with blood flow control in brain and heart and then assess the morphological and functional features of pericytes in these organs. We then focus on understanding the roles for pericytes in supporting neuronal and myocyte function through precision modulation of blood flow in these distinct contexts.

## CONTROL OF BLOOD FLOW IN BRAIN AND HEART

The angioarchitecture of the cerebral cortex consists of pial arteries on the brain’s surface, which branch at right angles, yielding functionally distinct penetrating arterioles that dive into the tissue (Figure 1). From here, an incredibly dense and tortuous capillary bed emerges with pericytes and their processes adorning most of the outer wall of this network. Given that the capillary bed composes ~90% of the brain vasculature by volume (22) and that these vessels are tightly interwoven with neuronal processes and cell bodies, the pericytes found here are ideally positioned to receive and process information on local neuronal activity and tune blood flow in response. Depending on their form and location within the capillary bed (see below), pericytes make differing contributions to this processing and help to solve several key challenges for blood delivery throughout the brain.

At the global level, the fixed volume of the skull imposes limitations on vascular dynamics and as such substantial changes in the volume of blood or cerebrospinal fluid can affect intracranial pressure and damage the brain (23, 24). Thus, changing global perfusion to the brain by dilating extensive portions of major feed arteries and arterioles (e.g., as might happen in working muscle) may adversely affect intracranial pressure and endanger neuronal function and therefore needs to be avoided. Consequently, the brain has evolved intricate mechanisms to focally modulate vessel diameter and blood flow, avoiding potentially damaging large changes in perfusion. It is now emerging that pericytes play a major role in this process.

At the local level, neuronal activity varies dramatically within individual cells and across populations. For example, pyramidal neurons discharge at about 1 Hz at rest and may increase their firing rate by more than an order of magnitude during activity (25–28), whereas fast-spiking interneurons from humans can fire in excess of 300 Hz (29). Increases in firing rate impose a huge increase in energy consumption (30), and the energy required to underpin this activity is primarily dedicated to ionic pumping mechanisms such as the sodium/potassium (Na^+^/K^+^) ATPase and the plasma membrane calcium (Ca^2+^) ATPase, which reverse the ionic fluxes that occur during action potentials and synaptic activity (30, 31). As the energy needed to fuel these processes is ultimately derived from the blood, this necessitates tight functional linkage between neuronal activity and increases in blood flow through the process of functional hyperemia, underpinned by the mechanisms of neurovascular coupling. This blood flow increase may also serve other important homeostatic functions, such as waste removal and temperature regulation. Accordingly, the brain has evolved a series of unique, layered, and redundant blood flow control mechanisms that span varied spatiotemporal scales and operate in concert to guarantee energy supply through orchestrated molecular activity that plays out across the multiple cell types of the neurovascular unit [endothelial cells (ECs) and smooth muscle cells (SMCs), pericytes, neurons, and astrocytes] (32). Pericytes sit at the center of this activity, nestled between astrocytic endfeet and the EC wall, and are thus ideally positioned to facilitate communication between the parenchyma and the vasculature to regulate local blood flow (33).

A distinct set of blood flow control problems accompany cardiac activity, and coronary blood flow control mechanisms (i.e., regulation of blood flow that the heart supplies to itself through the coronary arteries) have evolved to guarantee a continuous and extremely efficient supply of energy to support moment-to-moment heart work for an entire lifetime. Here too, it is now emerging that pericytes are intimately involved in these processes. Coronary blood flow varies with time depending on aortic pressure, myocardial extravascular pressure, and resistance to flow, which, in turn, are critically dependent on myocardial metabolism and neural and hormonal controls (for reviews, see 5, 7, 34, 35). These influences converge to regulate the contractile state of the small arterioles of the heart and their SMCs. The functional unit of blood flow regulation in heart is shown in Figure 3e. This functional unit embodies three sets of components that form the EMS system in heart. First, the ventricular myocytes are the primary metabolic sensors; second, the capillary pericytes, contractile pericytes, and end-arteriole smooth muscle cells are the primary blood flow regulators; and third, the local cECs are the primary electrical transmission elements in the local signal distribution network (10). We focus here on small vessel regulation in heart, as these vessels are more likely to receive direct input from local downstream pericytes and signals from adjacent ventricular myocytes, but we note that larger vessels also contribute to this process (35).

Coronary blood flow must be nearly perfectly matched to the metabolic demands of the heart at all times, which is evidenced by a near-maximal extraction of O_2_ from blood in the heart under all flow conditions. Blood appears to be directed to where there is metabolic need and is diverted away from regions that are replete with nutrients. The heart will adjust flow partially to compensate for local deficits where appropriate, a system that is somewhat analogous to control of blood flow in the brain by neurovascular coupling. However, the amount of O_2_ consumed by the heart and extracted from coronary blood is the highest of any organ per gram of tissue (36, 37). Put another way, the arterio-venous pO_2_ (partial pressure of oxygen) difference in heart is nearly always maximal, and the ratio of arterial pO_2_ to venous pO_2_ is maintained at around 1.6 to 1.7 unless the arterial pO_2_ falls to under 60% of its normal level (36, 37). It appears that a combination of the voltage sensitivity of SMC contractions, the electrical and contractile functions of pericytes, and their local signaling functions must work in tandem to continually and dynamically adjust blood flow through this system over a wide range of conditions. Nevertheless, precise, quantitative experiments are needed to accurately determine how pericytes in both of these contexts work, how they collate and distribute information, and how they act on it to control blood flow.

## THE PERICYTE CONTINUUM

**Figure 2:**
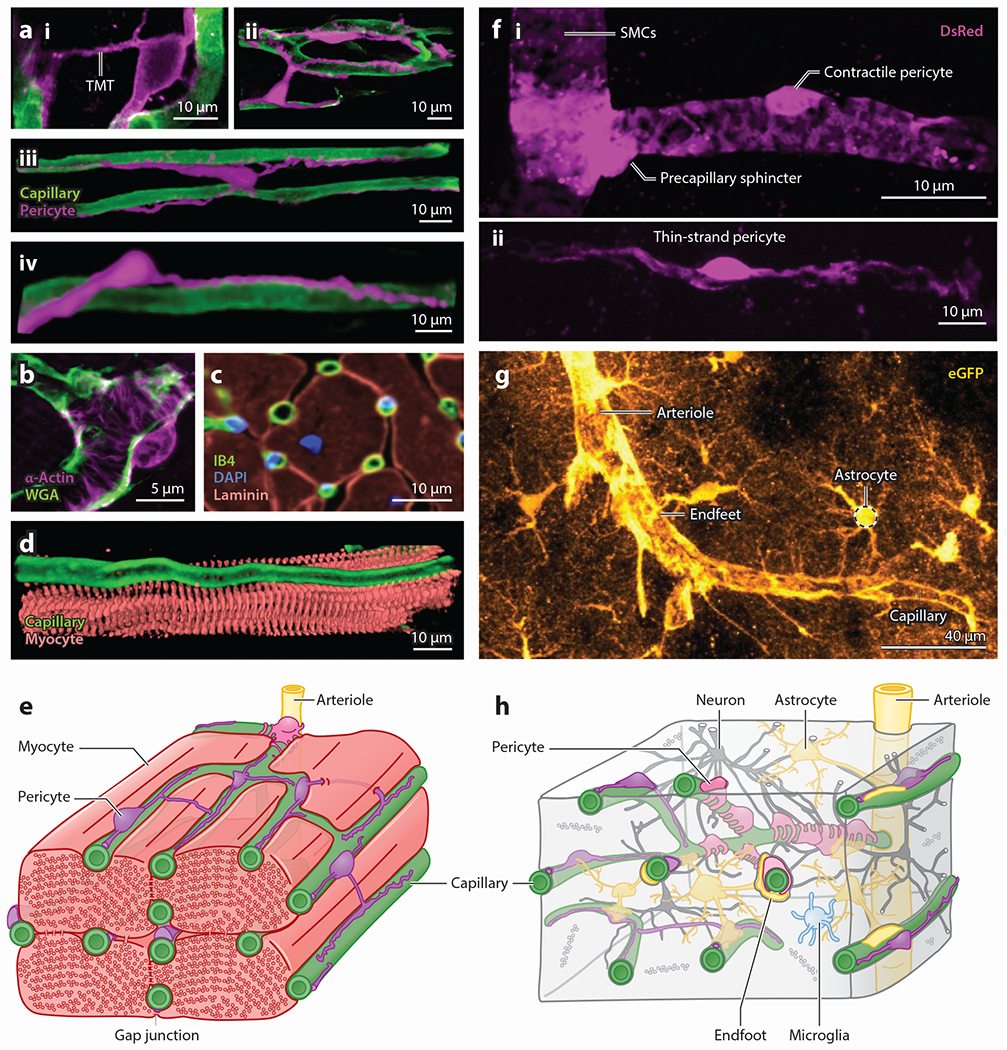
Pericytes in heart and brain microcirculation. (*a*) Confocal images showing the morphological diversity of capillary pericytes in heart. (*i*) A pericyte with a TMT leaping from one capillary to another, (*ii*) an intercapillary pericyte bridging one capillary to a capillary loop with its processes, (*iii*) an intercapillary pericyte bridging parallel capillaries with its cell body, and (*iv*) a capillary pericyte in heart. Magenta denotes NG2-DsRed and green FITC-WGA. (*b*) A contractile pericyte in heart. This confocal image shows an α-actin (*pink*)-positive pericyte wrapping around arteriole-capillary junctions (*green*; WGA) with thick processes. (*c*) Immunostaining image showing that each myocyte is surrounded and supplied by 4–6 capillaries. Panel adapted with permission from Reference 179; copyright 2014 Springer Nature. (*d*) Confocal image showing close contact between a myocyte (*pink*; actinin) and a capillary (*green;* WGA) that follows a furrow along the cardiac cell. (*e*) Diagram illustrating the mixed cell populations and the organization of capillaries (*green*), myocytes (*light red*), and pericytes (*pink* and *purple*) in heart. Each myocyte is surrounded by 4–6 interconnected, pericyte-embroidered capillaries. Capillaries in heart are easily recognizable by their parallel orientation with myocytes. Contractile pericytes wrap around arteriole-capillary junctions, a vantage from which they may exert profound influence over blood flow. (*f*) Detailed images showing morphological differences between (*i*) SMCs, the specialized precapillary sphincter at the border between arterioles and capillaries, contractile pericytes, and (*ii*) thin-strand pericytes of the brain. Pink denotes DsRed expressed under the NG2 promoter. (*g*) Expression of eGFP (*gold*) in astrocytes reveals the extensive endfoot coverage that these cells establish around both arterioles and capillaries. (*h*) Diagram detailing the mixed population of cells that surround the tortuous vascular network of the brain. Pericytes are nestled at the center of the neurovascular unit and are in close proximity with astrocytic endfeet, neurons, and microglial processes and directly contact the endothelium. Abbreviations: DAPI, 4′,6-diamidino-2-phenylindole; eGFP, enhanced green fluorescent protein; FITC, fluorescein isothiocyanate; IB4, isolectin B4; NG2, neuronal-glial antigen 2; SMC, smooth muscle cell; TMT, tunneling microtube; WGA, wheat germ agglutinin.

Ongoing debates surrounding the precise identity and classification of pericytes throughout the capillary bed require that we define both the varieties of these cells at the different levels of the vascular tree and in different organ systems and the criteria used to delineate the border between arterioles and capillaries. In the brain, extensive and ongoing work has been performed to carefully identify the morphological, functional and transcriptomic properties of pericytes throughout the vascular bed (Figure 2f). Arteries and arterioles can be distinguished from capillaries by two major features. (*a*) Arteries and arterioles secrete elastin (38, 39), a major component of the internal elastic lamina that can be stained to highlight these vessels. This elastin lining abruptly ends at the transition to the capillary bed (40) (Figure 1b). (*b*) Arteries and arterioles are suitably wide to allow the passage of red blood cells (RBCs) side by side, whereas capillaries are narrow enough to allow only for single-file RBC transit (Figure 1c). Accordingly, we define the initial capillary of a vascular network under consideration as the first to display a lack of elastin staining and to permit RBC passage in single file. Brain capillaries are referred to by branch order, so that the first capillary emerging from the arteriole is labeled as the first-order capillary. This numbering increases by one each time the capillaries branch as they extend deeper into the parenchyma, regardless of vessel diameter or orientation (Figure 1d). In the heart, the size and biochemical distinctions noted above hold true, but the ordered, sequential branching of capillaries rapidly breaks down as one examines the deeper capillary bed. Instead, capillaries here are much more elaborately and abundantly interconnected, with frequent anastomoses between vessels arrayed in near-parallel fashion around adjacent cardiac myocytes to form a looping, meshed network through which RBCs may take many potential paths (10, 41–43) (Figure 1e,f). In both systems, the capillary bed terminates when vessels unify at venules that pass deoxygenated blood and waste products out to larger veins (Figure 1d,e).

Arteries and arterioles are covered by SMCs that receive local electrical signals from their underlying arteriolar ECs and from downstream cECs. These SMCs enwrap the underlying cobblestone (44) arteriolar EC network concentrically, and the mechanisms that underlie their regulation have been extensively studied (Supplemental Box 1). SMCs are distinguished from bump-on-a-log, undulating pericytes by their concentric orientation and relatively flat, banded, fusiform morphology (45–48).

At the transition to the capillary bed, a specialized structure known as the precapillary sphincter has been identified, present at ~70% of all such branch points (49). Control of this juncture and the initial branches that follow is key to regulating perfusion of the capillary bed, mediated through the dynamic regulation of the contractile state of α-smooth muscle actin (α-SMA)-positive mural cells (49–51). Indeed, in the brain the first few capillary branches are covered by such contractile cells that actively regulate the diameter of the underlying vessel, which have been variably referred to as contractile pericytes (48, 52), SMCs (45, 53), ensheathing pericytes (20, 54), and hybrid cells (55). Here, we use the term contractile pericyte as, in our view, this best reflects the mounting evidence that these cells are functionally distinct from upstream SMCs, and this name helps to distinguish these cells from their less contractile counterparts further downstream. However, we emphasize that this is not an exclusive term. Rather, its use helps to underscore the key role of these cells in rapid diameter control of the underlying capillaries. Other pericyte types are also capable of contracting, but they appear to operate on slower timescales and have more subtle effects on diameter (56). Indeed, a major feature that contractile pericytes have in common with upstream SMCs is their expression of α-SMA (42, 48, 54, 57), and it is noteworthy that these cells cannot be distinguished from SMCs in genetic screens (58–60), likely as a result of their common lineage (61). However, mounting morphological and functional differences can be used to distinguish contractile pericytes from their SMC counterparts. Morphologically, contractile pericytes are distinguished by their bulging, ovoid cell bodies that give way to large finger-like projections that enwrap the underlying capillary (Figures 1a and 2b,f), covering up to about 95% of the outer surface of the vessel and giving the pericyte tight control over vessel diameter (46, 54). At the protein level, the Ca^2+^-binding protein calponin is detectable in SMCs but is not evident in contractile pericytes, and the same is true for polymerized microtubules (48). In contrast, contractile pericytes exhibit high levels of the intermediate filament desmin, aminopeptidase N (also known as CD13), the membrane chondroitin sulfate proteoglycan NG2, and platelet-derived growth factor receptor-β (PDGFRβ), which starkly contrasts with lower levels of these proteins in upstream SMCs (42, 57). Control of cytosolic Ca^2+^ is central to the regulation of the tone of the Ca^2+^-sensitive contractile apparatus in cardiac, skeletal, and smooth muscle (Supplemental Box 1). SMCs rely heavily on Ca^2+^ release through ryanodine receptors (RyRs) to regulate tone (62–64), whereas functional RyRs have not been detected in contractile pericytes (48, 65). Instead, inositol-1,4,5-trisphosphate (IP_3_) receptor–mediated intracellular Ca^2+^ signaling appears to play a prominent role in the contraction of these cells (48). It is anticipated that more differences between SMCs and contractile pericytes will emerge through further experimental efforts.

In the cortex and retina, the initial 3–4 branches of the capillaries emerging from the arteriole are covered by these contractile pericytes (Figure 2f,h), which play an important role in the branch-by-branch control of local blood flow. Immediately downstream of contractile pericytes on ~fourth-order capillaries in brain is a population of mesh pericytes that can be distinguished on the basis of their morphology, possessing a tangled network of processes, covering a lower fraction of the capillary surface, and expressing lower levels of α-SMA (54). Little is known of these cells, and the fact that a distinct transcriptomic signature to separate them from adjacent pericyte types has not been identified makes finding inroads for further detailed study challenging (66). Downstream of mesh pericytes, from approximately the fifth branch order and beyond, the capillaries are covered by thin-strand pericytes (Figure 2f,h). These cells express very low levels of α-SMA and extend long, thin filamentous processes away from the cell body that reach in some cases for hundreds of microns (54, 67, 68), taking on a diverse range of shapes and lengths.

Owing to the high density of capillaries in heart [~2,000–3,000 capillaries/mm^2^ (69)] and the distinct organization of both the capillaries and pericytes in this system, we use an alternate but related terminology to divide the pericytes found in heart into two types: contractile pericytes and capillary pericytes (42, 70–72). Based on our own and existing morphological observations using electron and confocal microscopy, we observe that α-SMA–containing pericytes are primarily seen at arteriolar-capillary junctions (see Figure 2b,e) in heart, which we term contractile pericytes. This name distinguishes them from the more abundant and again less contractile pericytes found deeper in the complex, anastomosing cardiac capillary bed, called capillary pericytes (42, 70, 71, 73, 74) (Figure 2a). We describe these subtypes in greater detail below. A complicating factor in drawing such a limited distinction is that cardiac pericytes lack cell- and subtype-specific biomarkers (a similar problem plagues the pericytes of the brain) that can be used for their unequivocal identification and classification. This issue highlights the need for an improved strategy for pericyte/SMC distinction, which might be achieved with a dual labeling system. For example, expressing one fluorophore under the control of the promoter of the elastin gene, *Eln*, and another fluorophore with separable spectral properties under the PDGFRβ gene, *Pdgfrb*, to distinguish elastin+/PDGFRβ+ SMCs from elastin−/PDGFRβ+ pericytes might be useful in this regard. However, in keeping with current strategies used to study brain pericytes, NG2 (encoded by the *Cspg4* gene) and PDGFRβ (*Pdgfrb*) alone are two markers that can be used to at least initially identify cardiac pericytes and reveal their physical structures and locations (10, 42, 43, 60, 75–77).

Cardiac contractile pericytes wrap around the components of the arteriolar-capillary junction (78) (Figure 2b), a positioning that enables them to exert powerful influence over local blood flow. Both contractile and capillary pericytes in heart appear to be capable of contracting to differing degrees (42), which aligns with recent findings for brain capillary pericytes (48, 52, 56). In the brain, upstream contractile pericytes contract more robustly and more quickly than their downstream thin-strand counterparts (48, 56). However, pericyte contraction dynamics have not been as thoroughly characterized in heart, regardless of the findings on fixed and nonperfused tissues indicating that pericytes might be involved in capillary constriction in ischemia-reperfusion (42); this area awaits further detailed study.

Pericytes on deeper heart capillaries are broadly similar to the thin-strand pericytes of the brain, but the lack of a reliably sequential vascular branching pattern that leads to an easily recognizable capillary hierarchy makes their identification based on branch order much more difficult (50). Indeed, the ovoid cell body of heart pericytes protrudes from the capillary wall with the classic bump-on-a-log appearance as seen in brain pericytes (79, 80). Primary processes from heart capillary pericytes extend from the cell body along the long axis of the underlying capillary tube, and secondary circumferential processes originate from these longitudinal stems and wrap at least partway around the vessel circumference, with an average coverage of about 11% of the abluminal vessel surface (79). Furthermore, cardiac pericytes are distinct from those in the brain by their variety and the fact that they frequently have rogue processes that appear to jump from one capillary to another by means of a large diameter (1 micron or more) appendage. This is called a tunneling microtube (Figure 2a) or TMT to distinguish it from the thinner interpericyte tunneling nanotubes, or IP-TNTs, found in the retina (41, 81, 82). These TMTs tunnel extensively between and around ventricular myocytes but with much larger tubes than TNTs. When a TMT lands on a target capillary, it often runs along that capillary for many tens of microns or more (Figure 2a), in contrast to TNTs, which typically terminate on other pericytes (82). We find that TMTs are particularly common in the epicardium and papillary muscles (Figure 2) and allow a pericyte to contact multiple stretches of capillary along its length. The leaps and branches of the TMTs in heart suggest an important signaling or coordinating aspect to their function that presumably complements pericyte contractile and hemodynamic control functions. An additional and possibly important feature of the TMTs is the direct contact with cardiac ventricular myocytes along their route, which may permit important signaling exchanges (Figure 2a,e). In the retina, ~30% of pericytes exude TNTs (82), and these are found traversing the parenchyma to connect pericytes of adjacent capillary beds, encountering a complex network of neuroglial processes on their way (Figure 2h). These processes appear to play a prominent role in blood flow regulation through Ca^2+^-dependent mechanisms (82). Clearly, both TMTs and TNTs represent fertile ground for further studies of pericyte contributions to blood flow control and integration of signaling throughout diverse circulatory beds (Supplemental Box 2).

The transcript profile of heart capillary pericytes is also similar to those of brain thin-strand pericytes, in that these cells robustly express *Rgs5* (which encodes the signal-transducing molecule regulator of G protein signaling 5) and the ATP-sensitive K^+^ (K_ATP_) channel subunits *Kcnj8* (Kir6.1) and *Abcc9* (SUR2), which can be used as markers and also suggest important functional contributions of these proteins (58–60, 76). Cardiac capillary pericytes also express *Acta2* (i.e., the contractile protein α-SMA) and *Tagln* (transgelin), but to a much lower degree than upstream SMCs and contractile pericytes (60).

## CAPILLARY ELECTRICAL SIGNALING: A BIOPHYSICAL LENS FOR UNDERSTANDING PERICYTE CONTRIBUTIONS TO BLOOD FLOW CONTROL

To understand how pericytes contribute to the control of blood flow in any organ system, it is important to establish how signaling is organized throughout the broader network in which the pericytes are embedded.

Prior work has established the electrical properties of arterioles and capillaries at the molecular, cellular, and systems levels (14, 18, 83–87). In arterioles, the endothelium is optimized to transmit electrical signals rapidly and efficiently over long distances via cell–cell gap junctions (44, 88, 89). In contrast, the overlying SMCs are less well coupled to one another (90) but receive input from the endothelium directly via specialized structures known as myoendothelial projections, also rich in gap junctions, which permit the lateral transfer of charge propagating through ECs into the SMC layer to exert its effects (91, 92).

**Figure 3:**
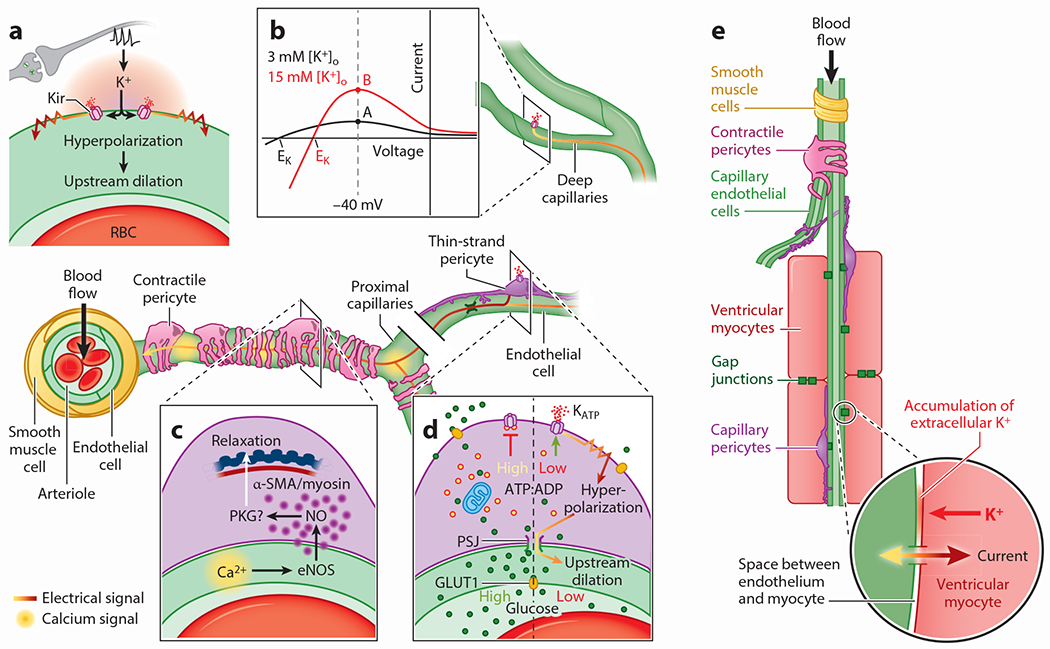
Blood flow control by electrical, Ca^2+^ and electro-metabolic signaling in brain and heart. (*Central image*) Top-down view of an arteriole enwrapped by SMCs and lined by ECs diving into the brain. On the proximal capillaries close to the arteriole are a population of pericytes (contractile) that express high α-SMA and rapidly regulate underlying vessel diameter. Deeper capillaries are covered by the cell bodies and processes of thin-strand pericytes that express low α-actin and regulate diameter over longer time frames and more modestly. Capillary blood flow is regulated by electrical (*a*, *b*), Ca^2+^ (*c*), and metabolic factors (*a*, *d*, *e*), all of which involve pericytes. (*a*) Neuronal activity elevates parenchymal K^+^ in the brain, which activates Kir channels to hyperpolarize the membrane, initiating electrical signals that propagate to upstream arterioles and contractile pericyte-covered branches, causing dilations. (*b*) Diagram illustrating the effect of elevated extracellular K^+^ on the current produced by Kir2.1 channels at −40 mV. Point A illustrates the relatively small current produced by Kir2.1 channels at −40 mV in cECs in the presence of extracellular [K^+^] of 3 mM which is typical for the brain parenchyma. Resting [K^+^] in the interstitial spaces of the heart and in blood plasma is higher. When extracellular [K^+^] around the cECs is elevated to 15 mM from 3 mM, there is an increase in outward current at −40 mV, as shown by point B. The increase in outward current hyperpolarizes the cECs to a potential negative to −40 mV. This counterintuitive effect occurs because of the upward and rightward shift in the IV relationship produced by the increase of [K^+^]_o_. Note that the Nernst potential for K^+^ (E_K_) moves to a more positive potential, but this is still negative to the resting potential of the cell. (*c*) Relaxation of contractile pericytes by capillary Ca^2+^ signals. Neuronal activity drives Ca^2+^ elevations in ECs of the proximal capillary bed, which couple to the generation of NO.As a gas, NO diffuses into pericytes and promotes relaxation of their contractile machinery, likely through a PKG–dependent mechanism that promotes membrane hyperpolarization and a fall in pericyte Ca^2+^. (*d*) Thin-strand pericytes act as metabolic sentinels in the brain that modulate EC electrical activity through K_ATP_ channel activity. In situations where the local availability of the key energy substrate glucose is reduced, the fall in intracellular ATP:ADP ratio increases K_ATP_ channel open probability, which promotes pericyte hyperpolarization. This hyperpolarization in turn is transmitted via pericyte PSJs into the underlying endothelium, where it modulates the electrical signaling mechanism described in panel *a.* (*e*) Local blood flow control by electro-metabolic signaling in heart. Local blood flow is controlled or regulated by electrically connected elements that include myocytes, capillaries, pericytes, and arterial SMCs, with myocytes as master controllers. As cardiac myocytes carry out work, ATP is consumed and ADP is produced and elevated. This leads to the opening of K_ATP_ channels in the cardiac myocytes and drives electro-metabolic signaling. K_ATP_ opening produces a time-averaged hyperpolarization of the cardiac action potential and the accumulation of K^+^ in the subspace between myocytes and capillaries. This leads to the hyperpolarization of the other elements in the network through gap junctions. In contrast, the elevation of extracellular K^+^ (through K_ATP_ and other K^+^ channels opening during repolarization) activates Kir2.1 in the cECs, resulting in cEC hyperpolarization and upstream contractile pericyte and smooth muscle relaxation. Abbreviations: ADP, adenosine diphosphate; α-SMA, alpha-smooth muscle actin; ATP, adenosine triphosphate; Ca^2+^, calcium; cEC, capillary endothelial cell; EC, endothelial cell; E_K_, potassium equilibrium potential; eNOS, endothelial nitric oxide synthase; GLUT1, glucose transporter 1; IV, current-voltage; K^+^, potassium; [K^+^]_o_, external potassium concentration; K_ATP_, ATP-sensitive potassium channel; Kir, inward rectifier potassium channel; NO, nitric oxide; PKG, protein kinase G; PSJ, peg-socket junction; SMC, smooth muscle cell.

Downstream of arterioles, we recently discovered that capillaries in both heart and brain employ a sophisticated electrical signaling system to fine-tune blood flow according to local metabolic needs (10, 14, 93, 94) (Figure 3). That this signaling paradigm operates in these two distinct capillary beds strongly suggests the possibility of its deployment in other organs too (e.g., kidney, liver, and lung), and further work is needed to directly explore these possibilities. The molecular lynchpin of this mechanism is the Kir2.1 K^+^ channel expressed by cECs. This channel is activated by increases in extracellular [K^+^] that leads to an outward, hyperpolarizing current (14, 15, 93) at the resting membrane potential of the capillary [approximately −35 mV in retina (95)], which is similar to the resting potential of arteriolar ECs and SMCs (Figure 3a,b). This resting membrane potential is relatively depolarized when compared to the K^+^ equilibrium potential (E_K_), which typically is around −100 mV under resting conditions in brain (Figure 3b). When external K^+^ increases as a by-product of activity in either heart (10) or brain (13, 96–101), Kir2.1 channels open to produce an outward, hyperpolarizing current, as shown in Figure 3. This may seem counterintuitive, as there has been a depolarizing shift in E_K_ due to the elevation of extracellular [K^+^]. However, E_K_ remains very negative to the resting membrane potential and therefore provides the outward driving force for K^+^ through the channel. Elevated extracellular [K^+^] opens the Kir2.1 channel by relieving the blockade of the pore by intracellular polyamines (15). Importantly, because the polyamine block of Kir2.1 is also voltage dependent, channel opening is further amplified by the generated hyperpolarization. This enables polyamines to relax from their pore occupancy and thereby permit robust K^+^ efflux (102), an action that drives membrane potential rapidly toward the new E_K_ (15). The activity of Kir2.1 is also dependent on the presence of phosphatidylinositol 4,5-bisphosphate (PIP_2_) (93, 103). While this action of Kir2.1 is dominant in brain, in heart it plays a smaller role (Supplemental Box 3).

These biophysical properties of Kir2.1 underpin the generation of hyperpolarizing electrical signals that propagate rapidly through the capillary network (>2 mm/s) and are passed via gap junctions from cell to cell to arrive upstream at the level of the arteriole (14). En route, these signals can relax upstream contractile pericytes of the transitional zone (a term referring to the initial 3–4 branches of the capillary bed that are covered by α-SMA-expressing contractile pericytes) (48, 57) and ultimately relax the SMCs of arterioles to control blood flow into the downstream capillary bed. At the level of arterioles, hyperpolarization is passed to SMCs via myoendothelial gap junctions and this in turn closes L-type voltage-gated Ca^2+^ channels, which causes a fall in cytosolic Ca^2+^, interrupting the Ca^2+^-dependent process of actin-myosin crossbridge cycling (Supplemental Box 1). This relaxes the smooth muscle contractile apparatus, which leads to vasodilation as a result of intravascular pressure acting on the vascular wall, ultimately driving an increase in blood flow into the downstream vessel. Contractile pericytes in the initial segments of the capillary bed relax through similar principles (48).

Several studies have noted the presence of peg-socket junctions formed between pericyte processes and cell bodies and underlying cECs (16, 17, 104, 105), which have been suggested to harbor gap junctions between these two cell types (79, 106). Together, this observation and the foregoing principles provide a framework with which to explore pericyte contributions to the control of brain blood flow: The intimate, interconnectedness of pericytes and cECs allows ongoing electrical signals to modulate pericyte membrane potential and thus intracellular signaling activity and contractile state, and pericytes will reciprocally influence electrical signaling in the capillaries to directly exert control of blood flow.

### Brain Pericyte Function Offers Clues to Heart Pericyte Function: Contractile Pericytes Direct Blood Toward Active Neurons

The recent intensive focus on brain pericyte function has allowed us to make rapid progress in our understanding of the intricate cellular interactions that control blood flow in this vascular bed, and this likely offers clues to understand pericyte contributions to blood flow control in other organs, including heart.

The transitional zone capillaries of the brain are a key site for blood flow regulation (Table 1). Similar to upstream SMCs, the contractile pericytes that adorn these branches exhibit around 40% myogenic tone (19) [i.e., under resting conditions they are preconstricted by ~40% from their maximally relaxed passive diameter, likely in response to intrinsic intravascular pressure sensing mechanisms (107) that remain to be defined]. From this basally constricted state, these cells can be relaxed by signals originating in the parenchyma or the underlying endothelium (14, 108, 109). As described above, capillaries employ an electrical signaling mechanism that is dependent on the activation of capillary Kir channels by neural activity-derived K^+^ that appears to relax not only upstream SMCs but also these transitional zone contractile pericytes (48), although this has not yet been studied exhaustively (Figure 3a,b).

Intimately linked with this electrical signaling mechanism, we also recently identified a highly localized Ca^2+^ signaling mechanism that regulates blood flow through capillaries covered in contractile pericytes (108) (Figure 3c). This mechanism depends on neurally driven G protein–coupled receptor signaling simultaneously engaging IP_3_ receptors and transient receptor potential vanilloid 4 channels (108, 110). In response to neuronal activity, the interplay between these molecular entities leads to a panoply of Ca^2+^ signals throughout the brain’s capillary bed. At the level of the transitional zone, these signals lead to the generation and release of nitric oxide (NO), a gaseous signaling molecule that rapidly diffuses to and relaxes overlying contractile pericytes. The Ca^2+^ events that precede NO generation are intimately dependent on capillary electrical signaling, which hyperpolarizes the membrane to increase the driving force for Ca^2+^ entry into ECs (108). Thus, the combined action of these electrical and Ca^2+^ signaling mechanisms regulates not only arteriolar tone but also pericyte contractile state to precisely tune blood flow into downstream capillaries. Electrical signaling operates to rapidly increase bulk blood flow into the capillaries, and complementary to this, Ca^2+^ signals operate on a highly localized scale to initiate and sustain relaxation of contractile pericytes overlying transitional zone capillaries and precisely direct incoming blood branch-by-branch toward the active neuronal population.

### Precision Matching of Energy Delivery to Brain and Heart Needs Through Capillary Pericyte Electrical Signaling

The contractile pericytes of the transitional zone are clearly capable of rapidly regulating capillary diameter by virtue of their expression of contractile α-SMA, a conclusion supported by data from several groups using a range of preparations and stimuli (Table 1). Thin-strand pericytes of the deeper capillary bed have been more controversial, but a recent state-of-the-art study by Shih and colleagues (56) provided clear evidence that these cells can indeed contract on the timescale of seconds, which is enough to influence blood flow through the underlying capillary. By expressing channelrhodopsin-2 in pericytes, this elegant study determined that stimulation of an individual thin-strand pericyte gradually produces a modest constriction that reduces the velocity of passing RBCs, providing direct evidence for modulation of capillary diameter by thin-strand pericytes and supporting and extending earlier observations (111). Interestingly, thin-strand pericyte-covered capillaries do not appear to dilate in response to neuronal activity (Table 1). Rather, sensory stimuli that increase blood flow appear to produce no change in the diameter of these capillaries. Thus, these cells do not seem to relax to cause dilation under conditions that produce substantial dilation of upstream arterioles to regulate blood flow. Can these cells control blood flow via other means?

Given the presence of peg-socket junctions between thin-strand pericyte processes and cell bodies and the underlying capillary ECs (17, 104, 105), we recently hypothesized that these may be specialized hubs around which signaling elements that mediate communication between ECs and pericytes organize to optimize the transfer of information between these two cell types. The presence of gap junctions here suggests that thin-strand pericytes may communicate with the underlying endothelium by generating signals to tune ongoing electrical activity and vice versa. To begin exploring this possibility, we surveyed a single-cell RNA sequencing data set (58, 59) to assess the relative expression of all ion channel genes in thin-strand pericytes of the brain (21). Strikingly, *Kcnj8*, which encodes the pore-forming subunit of the vascular K_ATP_ channel, accounts for approximately half of all ion channel gene expression in these cells. The auxiliary sulfonylurea subunit, SUR2, is also highly expressed by pericytes. This remarkably high expression of K_ATP_ subunits suggests an important role for these channels in pericyte physiology, and recent recordings have revealed large K_ATP_ currents in acutely isolated brain pericytes (alongside smaller currents also detectable in adjacent capillary ECs) (95).

In other tissues, such as pancreas and heart (10, 112–115), K_ATP_ channels couple changes in intracellular metabolism to electrical activity in the cell membrane. Thus, we reasoned that the high expression of K_ATP_ channels in thin-strand pericytes may position these cells as metabolic sentinels of the brain and that they may respond to local metabolic disturbances by generating hyperpolarization that can then be passed into the endothelium via peg-socket gap junctions to tune electrical signaling throughout the underlying capillary bed and shape blood flow (Figure 3d). We observed that delivery of K_ATP_ channel agonists produces a robust increase in arteriolar diameter in the cortex. In contrast, delivery of glibenclamide alone had no effect on vessel diameter. These observations suggest that there is a pool of functional, readily available K_ATP_ channels, but their basal activity does not contribute to basal vessel tone in vivo (19). This aligns with prior observations of K_ATP_ channels at the level of SMCs, which may be tonically inhibited by ATP or protein kinase C under resting conditions (116–118).

K_ATP_ channels are inhibited by intracellular ATP (119), so one possibility is that these channels are kept closed under resting conditions when pericyte intracellular ATP levels are relatively high and are subsequently engaged during metabolic activation of the cells as ADP levels rise. Indeed, native Kir6.1/SUR2-containing channels (120) have a high affinity for ATP, which exhibits an inhibitory action through a binding site on the pore-forming subunit with an IC_50_ of ~29–200 μM (114). In contrast, these channels also exhibit high sensitivity to ADP levels, which when bound by magnesium (Mg^2+^) ions, forming MgADP, bind to a site on the auxiliary sulfonylurea subunit to induce channel opening (120). This activating effect of MgADP exhibits an EC_50_ of ~16 μM in cells that do not express caveolin 1, and 96 μM in cells expressing this caveolae-forming protein (121), concentrations that represent the lower end of the physiological range of intracellular ADP (122–124). Given the high affinity of vascular K_ATP_ channels for ATP, it is unlikely that this will be depleted to a level that will substantially affect channel activity. Rather, the high level of ATP in the cell (~1–2 mM in other vascular cells) (125, 126) may keep these channels closed under basal conditions and, instead, rising ADP may be the major driver of metabolic activation of pericyte K_ATP_ channels. Critically, ATP/ADP levels in native pericytes have not yet been directly measured, and technologies and strategies are needed to measure these in real time in vivo and correlate these to readouts of K_ATP_ activity. Furthermore, whether the ADP:ATP ratio is directly influenced by physiological fluctuations in energy availability remains unclear; so far, the key experiments supporting this possibility make use of extreme metabolic conditions to elicit channel activity that are unlikely to occur in vivo (127, 128). These are key questions to address going forward.

Strikingly, we discovered that manipulation of energy substrate supply to pericytes evokes a pericyte K_ATP_ channel–dependent and EC Kir channel–dependent electrical signal that propagates to upstream arterioles and drives profound dilation to elicit an increase in blood flow to the underlying capillaries (19, 21). Specifically, we observe that progressively lowering bath glucose or inhibiting glucose transporter 1 (GLUT1) glucose transporters to lower local glucose in the brain elicits a digital electrical response from capillary pericytes. Indeed, a drop below ~1 mM [within the range of physiological parenchymal glucose (129–134)] leads to a profound K_ATP_ channel–mediated membrane hyperpolarization averaging ~15 mV. This K_ATP_-mediated electrical signal is likely fed via gap junctions into the underlying cEC syncytium to modulate ongoing electrical signaling and increase capillary blood flow (although it is also possible that K^+^ from K_ATP_ channel activity may also engage local EC Kir channels) (19). These data are consistent with a role for capillary pericytes as metabolic sentinels in the brain, sensing local depletions of metabolic reserves and responding through electrical signaling to increase blood flow to the affected region and replenish local energy supply, thereby protecting neuronal health and function (Figure 3d). Accordingly, pericytes appear to operate alongside certain neuronal populations (135) and astrocytes (136, 137) to monitor energy availability and enact homeostatic mechanisms to preserve a continuous supply of substrates. It is notable that capillary ECs also express functional K_ATP_ channels (95), which could also conceivably contribute to energy sensing. However, direct stimulation of capillary ECs with a K_ATP_ agonist had no effect on blood flow despite the same maneuver when applied to pericytes causing robust increases (19). These data thus support a predominant role for pericyte K_ATP_ channels in capillary blood flow control, and further experiments are needed to assess (*a*) whether this is the direct result of changes in pericyte ADP:ATP ratio and (*b*) whether a more subtle contribution from EC K_ATP_ channels can be detected.

Intriguingly, K_ATP_ channel activation may also promote GLUT1 translocation to the membrane (138). The molecular mechanism of this linkage is not known, but if this occurs in capillaries in vivo, it could further boost glucose uptake during K_ATP_ signaling for a blood flow increase. Another interesting relationship between these two proteins with similar ramifications is that both have binding sites for ATP. While free ATP inhibits K_ATP_ channels, as discussed above, binding of ATP at the GLUT1 nucleotide binding site increases glucose binding capacity but inhibits its import into the cytosol. This ATP modulation of sugar transport is competitively inhibited by AMP and ADP (139–141) and, accordingly, changes in the intracellular energetic landscape that open K_ATP_ (i.e., increasing the ADP:ATP ratio) could also potentially promote high-capacity glucose import by relieving ATP inhibition of GLUT1. Further experiments are needed to examine these possibilities.

Coupling of local energy fluctuations to blood flow is also extremely important in heart, where arteriolar membrane potential is controlled by EMS throughout the vasculature that supplies blood to a local region of myocardium (10, 142). Although this is an area of active research, we propose that EMS is the primary controller of membrane potential in cardiac small vessels, including local SMCs (9, 10) (Figure 3e). This is particularly easy to conceptualize when considering a region of the heart working hard to pump blood. This pressure-volume work is carried out as local regions of ventricular myocytes consume ATP to produce a decline in [ATP] and rise in [ADP] within the ventricular myocytes. Together, these changes in adenine nucleotides activate the robustly expressed cardiac K_ATP_ channel [composed in this case of Kir6.2/SUR2A subunits (114, 115), which produces a time-averaged hyperpolarization of the ventricular myocyte (10, 35, 143, 144)]. This hyperpolarization produces a negative shift in the membrane voltage of the local vascular tree through the very sparse number of gap junctions that connect cardiac ventricular myocytes to cECs (Figures 2e and 3a,e; Supplemental Box 3). Accordingly, hyperpolarization within the cECs spreads through the vasculature where it may promote hyperpolarizations of local pericytes and upstream SMCs, analogous to the situation in the brain vasculature, and thus leads to their relaxation to promote an increase in blood flow to the cardiac ventricular myocytes (10, 87). In the heart vasculature, there is also a secondary contribution to the regulation of membrane potential and thus of blood flow through the local capillaries by Kir2.1 channels (14, 15, 35). Indeed, capillary Kir2.1 activity is engaged by external K^+^ or hyperpolarization during EMS to support hyperpolarization of upstream pericytes and SMCs. This effect can be achieved through K_ATP_ channel activity in ventricular myocytes, which when activated will directly inject hyperpolarizing current into cECs and also elevate external K^+^ ([K^+^]_o_) to activate Kir2.1 (14, 145). Importantly, pinacidil (a K_ATP_ channel-opening drug) produces vasodilation in heart even when the K_ATP_ channels in pericytes, cECs, and SMCs are knocked out (10). Thus, EMS can work alone to dilate cardiac small vessels when driven solely by ventricular myocytes but can also recruit help from Kir2.1-mediated electrical signaling. Ongoing investigations seek to identify how each mechanism contributes to the overall small vessel blood flow regulation.

Together, these collected findings represent state-of-the-art information on how brain and heart tissues use known and emerging features of the microcirculation and characteristics of small vessel elements to regulate blood flow through metabolic control. At the small vessel level, several mechanisms use metabolic cues and measures to provide a local signal to regulate flow. EMS, the umbrella term for these mechanisms, uses metabolic sensors to generate signals to increase blood flow in response to metabolic needs. As ventricular myocytes or brain pericytes consume ATP, cytosolic [ATP] declines and cytosolic [ADP] rises. Together, these concentration changes activate K_ATP_ channels to hyperpolarize the relevant cells (i.e., ventricular myocytes in heart or pericytes in brain), which then inject hyperpolarizing current into cECs. This hyperpolarization of the cECs then spreads upstream to contractile pericytes and arteriolar SMCs producing relaxation and increasing local blood flow. This electrical activity, including the opening of K_ATP_ channels, also increases parenchymal [K^+^], which further hyperpolarizes cECs, increasing blood flow to provide the needed O_2_ and nutrients that are critical for ongoing healthy tissue function.

## PERICYTE DYSFUNCTION AS A KEY CONTRIBUTOR TO ORGAN FAILURE STATES

### Brain

Pericytes in the brain are ideally positioned to impact blood flow, regulate the tightness of the blood–brain barrier, and influence angiogenesis (146). Dysfunction of pericytes may be pivotal in a range of systemic diseases and central to the development of neurodegenerative conditions and diverse forms of brain failure. Indeed, small vessel or microvascular dysfunction has been increasingly implicated in a range of brain disorders (147), and brain pericytes are especially sensitive to the hypoxia that typically accompanies these (148, 149). Two major types of brain vasculopathies involve critical pericyte loss—the inherited condition of cerebral autosomal dominant arteriopathy with subcortical infarcts and leukoencephalopathy (CADASIL), a form of hereditary stroke that causes small vessel disease of the brain (150), and the age-related disorder Alzheimer’s disease. In both cases, early pericyte degeneration is observed (151–153), resulting in downstream vascular disturbances such as blood–brain barrier leakage and dysregulated capillary hemodynamics that likely contribute to the development of cerebral hypoperfusion and cognitive decline (154, 155). CADASIL is caused by missense mutations in the *NOTCH3* gene, which pericytes heavily express, and the ensuing damage to pericytes is a key pathogenic feature of the disease (156). Alongside the loss of peg-socket connections between pericytes and ECs (157), pericyte deficiency by itself also reconfigures the molecular machinery of ECs (158), which could further hamper electrical communication throughout the vascular network and result in neuronal injury due to a mismatch of activity and energy supply. Vascular changes are also possibly the earliest detectable pathological consequence of Alzheimer’s disease (159). Our recent identification of pericytes as metabolic sentinels (19) suggests that disruption of this role may contribute to vascular dysfunction underlying these brain disorders, particularly those such as Alzheimer’s disease, where glucose is profoundly dysregulated (160) and the expression of pericyte K_ATP_ channels is decreased (161). Further study is needed to examine these possibilities.

### Heart

Pericyte loss or dysfunction has been discovered in many diseases that affect small vessel and cardiovascular function or related pathological and pharmacological animal models. The affected systemic diseases include heart failure (77), diabetes (162, 163), and sepsis (164, 165), and similar observations have been made in Notch3 or PDGFB-PDGFRβ knockout mice (75, 164, 166–168). They may act primarily on pericytes (e.g., PDGFB-PDGFRβ knockout) and underlie microvascular dysfunction (such as increased microvascular permeability and leakage), while others may produce dysfunction secondary to capillary loss or inflammation or other damage. The phenotypes caused by pericyte loss or dysfunction may vary depending on whether the loss is temporary or permanent, localized or generalized. For example, general and permanent pericyte loss that is caused by PDGFB-PDGFRβ deletion results in perinatal death with cardiac defects, dilated heart failure, systemic vasodilation, and edema (169, 170). In contrast, transient and localized ablation of pericytes causes only the attached capillary to dilate (67, 170). These pathological phenotypes suggest an important cardiovascular role for pericytes, as does the complex and elegant anatomy presented in this review. However, ongoing and future studies are needed to determine how pericyte loss or damage affects the heart and how they interfere with the regulation of blood flow.

Another reported feature of pericyte function in heart is its plasticity and functional transition under physiological or pathological conditions. During development, pericytes can differentiate into coronary SMCs (171). Cardiac pericytes have also been shown to exhibit properties of mesenchymal stem/stromal cells with angiogenetic potential under pathological conditions (172). Thus there appears to be a pericyte plasticity that may be activated in heart failure, myocardial infarction, and hypertrophy, with some pericytes turning into fibroblasts and others into myocytes (172–174), regardless of changes in pericyte number (162, 175). However, recent work (176) showed that endogenous pericytes retain their properties during aging and in different pathological settings, suggesting that pericyte plasticity observed in vitro or after transplantation in vivo may be due to artificial cell manipulation (176). Therefore, it is necessary to further evaluate the links between changes in pericyte function and cardiac dysfunction by longitudinal intravital imaging of the heart. Additionally, pericytes appear to have a role in ischemic coronary no-reflow, altered mechanotransduction, fibrosis, and angiogenesis in heart disease (42, 74, 77, 172, 175, 177, 178). It is thus clear that pericytes contribute to the cell biology of the heart in diverse and unexpected ways, and extensive additional research is needed to address exactly what cardiac pericytes do and understand how they do it.

## KEY QUESTIONS AND FUTURE DIRECTIONS

It is an exciting time to study pericyte physiology and blood flow control. A confluence of recent technical advances, with more on the horizon, promise to yield further insights into the nature of signaling throughout the vasculature and are expected to dramatically reshape our view of pericytes, their signaling roles, and the spatiotemporal extent of their signaling processes. Below, we outline the key issues in broad categories that we think are most important to address in the near term, which are key to advancing our understanding most rapidly.

### Developing a Full Understanding of the Mechanisms of Pericyte Contraction

To fully understand pericyte control of blood flow, we need a quantitative understanding of the signals that regulate contraction in these cells. This must start with measurements of the time course of pericyte contractions and relaxations with physiologically relevant stimulations in heart and brain and other tissues. How do these pericyte changes affect capillary diameter and flow? Do they also affect pericyte extensions, pericyte processes, and TNTs/TMTs? Reciprocally, TNTs/TMTs may also contribute to pericyte contractions, adding an interesting dimensionality that is not available to other mural cells that lack these elaborations (82). A current top priority, which is within experimental reach, is to determine whether and how pericyte membrane potential affects contractile state. Another key question is how does intracellular Ca^2+^ ([Ca^2+^]_i_) contribute to the control of pericyte contractile state? Given the expression of several voltage-dependent K^+^ and Ca^2+^ genes in pericytes (21), it is likely that these parameters are inextricably linked. Addressing these questions and establishing the relative contributions of specific pericyte types to blood flow control are critical next steps that are already underway.

### Understanding the Nature of Pericyte Signaling Mechanisms that Generate Electrical Signals

A further critical area of investigation are the mechanisms that pericytes deploy to generate electrical signals of their own. The spatial spread of electrical signals originating in pericytes has been explored to some extent in elegant ex vivo studies (18), and a major next step is to test the translatability of these findings in the in vivo context. Key unaddressed questions are whether pericytes and ECs are so well coupled that their membrane potentials fluctuate in sync, or whether coupling is more muted to allow independent changes of membrane potential that influence the adjacent cells’ activity more subtly. Additionally, do pericytes sculpt signaling through the underlying ECs by acting as signal boosters for ongoing electrical signals, or alternatively as current sinks that dampen electrical signaling? And, more broadly, how does EMS in heart and brain affect the quantitative signals to and from pericytes? Through their unique sensing and signaling capabilities, pericytes could imbue capillaries with sensitivity to shifts in their local environment that they would otherwise not possess, allowing electrical responses to a broader range of cues. Thus, elucidating the roles played by pericyte and endothelial cell ion channels and G protein–coupled receptors will fill major gaps in our current knowledge. This should include full analysis of the regulation of K_ATP_ channels in both cell types. Also important is the delineation of roles for the endo/sarcoplasmic reticulum, sarco-endoplasmic reticulum Ca^2+^ ATPase pumps, and IP_3_Rs and RyRs within pericytes. Difficult to study, but also of central importance, is the nature of gap junction coupling between pericytes and ECs. Indeed, pericytes and ECs express a range of gap junction genes (21), which forms a take-off point for more detailed studies. Precisely determining which ion channels and receptors are functionally expressed and at what levels in each type of pericyte across organ systems, as well as the signaling mechanisms that drive their activity, thus represents a major undertaking for the field that will greatly inform our models of how and under what circumstances pericytes generate electrical signals and their contributions to blood flow control.

### Elucidating the Roles of TNTs and TMTs and Their Distribution Throughout the Body

TNTs in brain and the TMTs in heart likely sense unique information that is inaccessible to processes situated on the vasculature. Determining whether this is the case and how this information affects pericyte behavior and action are other exciting areas for further study. We should aim to establish the mechanisms of communication through these processes between cells. There are also key developmental questions that arise from our current data, such as when these processes develop and what directs the growth of these appendages and the organization of their attachments. These projections could also provide a structural scaffold for potential angiogenesis to reshape the physical branching of the anastomosing capillary network, both during development and in adult tissue. Understanding these pericyte processes may be key to unlocking a full understanding of how electrical and chemical signaling is organized in vascular networks to control blood flow across complicated biological terrain.

## SUMMARY AND CONCLUSIONS

Here, we present newly developed information on how blood flow is controlled in brain and heart, with a focus on pericytes in small vessels. SMCs, precapillary sphincters, and contractile pericytes are key controllers of local blood flow and are regulated in both heart and brain by electrical signals distributed by the capillaries to contractile elements through gap junctions. This permits upstream regulation of blood flow by tissues in metabolic need. The primary metabolic sensors in heart are the cardiac myocytes (ventricular and atrial) through the sensitivity of the K_ATP_ channels in their sarcolemmal membranes to metabolic stress. Hyperpolarizing electrical signals generated in the myocytes by low [ATP]_i_ and elevated [ADP]_i_ are passed to the cECs through gap junctions and form the basis of EMS in heart. In parallel to this process, thin-strand pericytes in brain act as local metabolic sentinels, which monitor energy substrate availability in their vicinity and respond to decreases in energy abundance with K_ATP_ channel activation, which modulates capillary electrical signaling to control blood flow and protect ongoing neuronal health and function. In addition, parenchymal [K^+^]—a primary by-product of metabolic activity in brain—is sensed by cECs in both heart and brain and exploits the properties of the Kir2.1 channels in the plasma membrane that are hyperpolarized by its local elevation. Additional EMS mechanisms in brain are likely to occur and are a topic of great interest that is being pursued foremost in pericytes, among other cells.

The investigations spawned by recent exciting findings related to pericyte biology and the control of blood flow centered in heart and brain enable new hypotheses and detailed studies of the mechanisms of blood flow regulation in small vessels. This work seeks to address the key questions outlined above and will be greatly advanced through full exploitation of at least the following four technical methods. (*a*) Optogenetic tools are needed to depolarize or hyperpolarize specific cells in the signaling chain between metabolically active elements (neurons, pericytes, and cardiac myocytes) and the cells that directly control blood flow. Thus, an exciting target includes the cECs in addition to pericytes themselves. (*b*) Fluorescence signaling reporters expressed in cells that regulate blood flow will be remarkably helpful. Thus, GCaMP8 (a state-of-the-art high signal-to-noise [Ca^2+^] reporter) should be expressed as a transgene in pericytes or SMCs to indicate how contractile cells respond to an initiating electrical signal. Moreover, use of modern, easy to implement, genetically encoded voltage sensors in the vasculature would enable the study of signal propagation throughout the vasculature with unprecedented detail. (*c*) Fluorescence lifetime reporters expressed in transgenic form to measure [ATP] in a targeted cell would also be exceedingly useful. Surmounting this technical challenge will enable us to answer questions related to energy balance that span many fields. (*d*) Multiphoton imaging is already being extensively used in brain to carry out such fluorescence experiments in living animals through long-term cranial windows. Although investigations in brain are ahead of those in heart because of the difficulty in carrying out such experiments in moving tissue, progress is being made in both areas, and the prospects are great for further important advances in both tissues.

## Supplementary Material

Supplementary Boxes 1-3

## Figures and Tables

**Table 1:** Brain pericyte contractility. A survey of data assessing whether pericytes covering the brain vasculature are able to contract and relax and over what timescale contractile operations occur

Branch order	Cell type	Reference	Preparation	Contractile stimulus	Diameter change (% versus baseline)	Onset time (time to peak)
0	SMCs	180	Parenchymal arteriole myography; mouse	Physiological pressure U46619 Caffeine	Constriction	Seconds
		181	Parenchymal arteriole myography; rat	Physiological pressure NOS block	Constriction	No data
		182	Parenchymal arteriole myography; mouse	Physiological pressure Purines	Constriction	Seconds
		56	In vivo cranial window	Optogenetic activation of ChR2	Constriction (40%)	Seconds (~25 s)
		45	In vivo cranial window	Optogenetic activation of ChR2	Constriction (~20%)	<1 s (> 15 s)
				Sensory stimulation	Dilation (~10%)	~2 s (~40 s)
		49	In vivo cranial window	Sensory stimulation	Dilation (~11%)	~1 s (~18 s)
		183	In vivo cranial window	Sensory stimulation	Dilation (~15%)	~1 s (~5 s)
		52	In vivo cranial window	Sensory stimulation	Dilation (~10%)	~3 s (~12 s)
1	Contractile pericytes	49	In vivo cranial window	500 mM K^+^	Constriction (~25%)	Seconds (30 s)
				Sensory stimulation	Dilation (~16%)	~ 1 s (~ 15 s)
		183	In vivo cranial window; vessels <50 μm from PA	Sensory stimulation	Dilation (~30%)	~ 1 s (~4 s)
		52	In vivo cranial window	Sensory stimulation	Dilation (~14%)	~ 1 s (~6 s)
1–4	Contractile pericytes	183	In vivo cranial window; vessels >50 μm from PA	Sensory stimulation	Dilation (10%)	~1 s (~5 s)
		45	In vivo cranial window	Optogenetic activation of ChR2	Constriction (~ 12%)	<1 s (> 15 s)
				Sensory stimulation (vessels with α-actin)	Dilation (~10%)	~2 s (~40 s)
		56	In vivo cranial window	Optogenetic activation of ChR2	Constriction (20%)	Seconds (>1 min)
5+	Thin-strand pericytes	56	In vivo cranial window	Optogenetic activation of ChR2	Constriction (19%)	Seconds (>1 min)
		111	Acute brain slices	U46619	Constriction (25%)	Seconds (>2 min)
			In vivo cranial window	U46619	Constriction (17%)	No data
				Sensory stimulation	No change	Not applicable
		183	In vivo cranial window	Sensory stimulation	No change	Not applicable

Abbreviations: ChR2, channel rhodopsin 2; K^+^, potassium; NOS, nitric oxide synthase; PA, penetrating arteriole; SMC, smooth muscle cell.
