# Supplementary material for: Pericytes and the Control of Blood Flow in Brain and Heart
Source: Annu Rev Physiol. Author manuscript; Available in PMC 2023 Jun 20. (PMC10280497; doi:10.1146/annurev-physiol-031522-034807)
Supplement: Supplementary Boxes 1-3 [file NIHMS1877508-supplement-Supplementary_Boxes_1-3.pdf]

SUPPLEMENTAL BOXES

SUPPLEMENTAL BOX 1. BASIC MECHANISMS CONTROLLING SMOOTH MUSCLE CONTRACTION

Ca^{2+} is central to small vessel diameter regulation, and therefore blood flow control, in all organs. Small arteries and arterioles contract when cell-wide cytosolic Ca^{2+} in their SMCs rises, which is a direct result of SMC depolarization which increases Ca^{2+} entry through voltage-dependent Ca^{2+} channels (VDCCs) (1, 2). Conversely, SMCs relax when they are hyperpolarized, which closes VDCCs causing a fall in global intracellular Ca^{2+}, preceding vessel dilation due to intravascular pressure pushing out on the vessel wall.

Within each SMC, relaxation also occurs when subcellular microdomain Ca^{2+} elevations that achieve micromolar local concentrations (known as Ca^{2+} sparks) occur. These are the result of the opening of a small cluster of RyRs on the sarcoplasmic reticulum, release through which elevates Ca^{2+} locally to activate adjacent large-conductance Ca^{2+}-activated K^+ channels (BK channels) in the sarcolemma (3, 4). This produces a brief, profound SMC membrane hyperpolarization referred to as a spontaneous transient outward current (STOC) which underlies the closing of sarcolemmal Ca^{2+} channels locally within the affected cell(s), driving down global Ca^{2+} and promoting the disengagement of crossbridge cycling (3, 5). While this global versus local Ca^{2+} signaling may seem paradoxical, an explanation is readily available. The global elevation of Ca^{2+} causes contraction through its interaction with calmodulin to stimulate myosin light chain kinase to drive crossbridge cycling. The spatially limited, highly localized subcellular Ca^{2+} sparks are too small and brief to cause a significant global increase of Ca^{2+} and thus instead causes local hyperpolarization and cellular relaxation (3, 5).

Supplemental Material: Annu. Rev. Physiol. 2023. 85:137-164
https://doi.org/10.1146/annurev-physiol-031522-034807
Pericytes and the Control of Blood Flow in Brain and Heart
Longden, Zhao, Hariharan, and Lederer

SUPPLEMENTAL BOX 2. INTER-PERICYTE TUNNELING NANOTUBES AND BLOOD FLOW CONTROL

Recently discovered inter-pericyte tunneling nanotubes (IP-TNTs) are described as thin, tunneling processes connecting the pericyte soma of one capillary to a process of another pericyte on a distinct capillary (6). These provocative structures are thought to be responsible for modulating blood flow and neurovascular coupling in the retinal capillary bed (6), and appear to be susceptible to pathological stresses (7). How these structures and the connections they form contribute to blood flow control in brain and other organ systems is an important topic of active investigation. In brain, recent evidence now points towards the existence of an abundant array of TNTs. For example, thin bridges consisting of nanotubes connecting pericyte somas and basement membranes between adjacent, neighboring capillaries in the human temporal cortex have been observed (8). In heart, these pericyte appendages are larger and hence we have dubbed these tunneling *micro*tubes or TMTs, which are also plentiful. Here, we show examples from heart in which we observe that microtubes extending from the pericytes are bigger and wend their way along capillaries for between ~20 and 40 microns, traveling across junctions around contracting ventricular myocytes then along adjacent capillaries (9, 10) (**Fig. 2**).

Comprehensive investigations of these tubular structures are needed to develop our understanding of their function, to determine how they behave along their course through the tissue, and to characterize their mechanisms of signaling. One would also seek to characterize similarities and differences in the brain and heart and determine shared versus contrasting features. How do these structures change during development? Are they capable of being formed *de novo* in times of stress or special need? And how is their function regulated? Since TNTs have traveling mitochondria within the nanotubes and exhibit bidirectional Ca^{2+} waves (6), we also ask what biophysical mechanisms govern the underlying processes of these phenomena. In particular, investigations into how TNT Ca^{2+} waves contribute to chemical and electrical signaling in capillary beds warrants further close attention. Further important questions focus on the nature of these pericyte-to-pericyte connections – are these akin to bridges between capillary segments originating from the same arteriolar network or do they connect two disparate networks? In the first scenario, it is possible to envision TNTs as electrical signaling shortcuts that bypass a tortuous capillary network, allowing for rapid and efficient conduction of signals along the path of least resistance to increase the speed of electrical communication. Also, in brain at least, it is more likely that TNTs connect two distinct capillary networks, given the more obvious zonal and hierarchical capillary structure. If so, the TNTs in brain may assist in redirecting blood flow towards a more active region. Indeed, light stimulation elicits simultaneous but opposite responses from capillaries connected by an IP-TNTs in the retina (6). In the brain, the interactions of pericytes with microglia or neuronal synapses are limited by the close positioning of astrocytic endfeet around pericytes. The long tubes that make up TNTs traverse through the tissue, and are therefore well-positioned to overcome this physical limitation imposed by endfeet to allow input from or signaling to other cells of the parenchyma with which they come into direct contact. Further studies will undoubtedly improve our understanding around these concepts and reveal important contributions of these remarkable novel structures.

SUPPLEMENTAL BOX 3. GAP JUNCTIONS IN HEART

Heart muscle has a huge array of gap junctions (GJs) electrically connecting ventricular myocytes end-to-end, as well as a moderate number of lateral gap junctions (11). Importantly, however, more gap junctions are not necessarily better. For example, the pacemaker of the heart is made possible by a balance of a sparse set of gap junctions combined with the specialized electrical properties of the sinoatrial node (SAN) cells involved. The SAN cells have intrinsic pacemaker activity from which the heartbeat originates, and the SAN cells are only able to pass on this electrical signal to the atrial myocytes when there are very few gap junctions between them. This way SAN cells can influence the electrical signal of their neighboring atrial myocytes without the atrial myocytes excessively dominating the electrical activity of the SAN cells. The SAN cells do this by injecting current into their immediately adjacent neighboring atrial myocytes through gap junctions to depolarize them (12). This is a tricky handoff of the heartbeat to enable the signal to spread to the rest of the heart. The SAN cells must inject just enough current through a *sparse* number of gap junctions to initiate the propagating action potential in the adjoining atrial cells without excessive "back-signaling" from them to the SAN cells. There is thus a "sweet spot" that must be found and maintained as a life-long activity (13). If there are too many gap junctions, the atrial myocytes will voltage clamp (i.e. control the voltage) of the SAN cells and the SAN cells will not be able to control the successful initiation and maintenance of heart rate. This example illustrates why very few gap junctions are likely to connect ventricular myocytes to cECs to produce electro-metabolic signaling (EMS, **Fig.3*e***) in heart. For ventricular myocytes to influence the resting potential of cECs and the nearby pericytes and smooth muscle cells, the myocyte action potentials (APs) need to be low-pass filtered and smoothed (9). By connecting the ventricular myocytes to the cECs through very few gap junctions, this can be achieved. Using this method, the metabolic status of the myocytes will modify their APs and through a low-pass filtering effect will influence the resting membrane potential of the cECs and thereby affect the contractile state of nearby connected pericytes and upstream arteriolar SMCs.

REFERENCES

1. Berridge MJ. 2008. Smooth muscle cell calcium activation mechanisms. *J. Physiol.* 586(21):5047–61
2. Nelson MT, Patlak JB, Worley JF, Standen NB. 1990. Calcium channels, potassium channels, and voltage dependence of arterial smooth muscle tone. *Am. J. Physiol. Cell Physiol.* 259(1):C3–18
3. Nelson MT, Cheng H, Rubart M, Santana LF, Bonev AD, et al. 1995. Relaxation of arterial smooth muscle by calcium sparks. *Science.* 270(5236):633–37
4. Jaggar JH, Porter VA, Jonathan Lederer W, Nelson MT. 2000. Calcium sparks in smooth muscle. *Am. J. Physiol. Cell Physiol.* 278(2 47-2):
5. Cheng H, Lederer WJ. 2008. Calcium sparks. *Physiol. Rev.* 88(4):1491–1545
6. Alarcon-Martinez L, Villafranca-Baughman D, Quintero H, Kacerovsky JB, Dotigny F, et al. 2020. Interpericyte tunnelling nanotubes regulate neurovascular coupling. *Nature.* 585(7823):91–95
7. Alarcon-Martinez L, Shiga Y, Villafranca-Baughman D, Belforte N, Quintero H, et al. 2022. Pericyte dysfunction and loss of interpericyte tunneling nanotubes promote neurovascular deficits in glaucoma. *Proc. Natl. Acad. Sci. U. S. A.* 119(7): e2110329119
8. Shapson-Coe A, Januszewski M, Berger DR, Pope A, Wu Y, et al. 2021. A connectomic study of a petascale fragment of human cerebral cortex. *bioRxiv*. 2021.05.29.446289
9. Zhao G, Joca HC, Nelson MT, Lederer WJ. 2020. ATP- And voltage-dependent electro-metabolic signaling regulates blood flow in heart. *Proc. Natl. Acad. Sci. U. S. A.* 117(13):7461–70
10. Zhao G, Joca HC, Lederer WJ. 2020. Dynamic Measurement and Imaging of Capillaries, Arterioles, and Pericytes in Mouse Heart. *J Vis Exp*
11. Nielsen MS, Axelsen LN, Sorgen PL, Verma V, Delmar M, Holstein-Rathlou NH. 2012. Gap junctions. *Compr. Physiol.* 2(3):1981–2035
12. Jansen JA, van Veen TAB, de Bakker JMT, van Rijen HVM. 2010. Cardiac connexins and impulse propagation. *J. Mol. Cell. Cardiol.* 48(1):76–82
13. Rohr S, Kucera JP, Fast VG, Kléber AG. 1997. Paradoxical improvement of impulse conduction in cardiac tissue by partial cellular uncoupling. *Science.* 275(5301):841–44
